# Cyclic GMP and PKG Signaling in Heart Failure

**DOI:** 10.3389/fphar.2022.792798

**Published:** 2022-04-11

**Authors:** Genri Numata, Eiki Takimoto

**Affiliations:** ^1^ Department of Cardiovascular Medicine, The University of Tokyo Hospital, Tokyo, Japan; ^2^ Department of Advanced Translational Research and Medicine in Management of Pulmonary Hypertension, The University of Tokyo Hospital, Tokyo, Japan; ^3^ Division of Cardiology, Department of Medicine, The Johns Hopkins Medical Institutions, Baltimore, MD, United States

**Keywords:** NO, SGC, NPR, PGC, cGMP, PKG

## Abstract

Cyclic guanosine monophosphate (cGMP), produced by guanylate cyclase (GC), activates protein kinase G (PKG) and regulates cardiac remodeling. cGMP/PKG signal is activated by two intrinsic pathways: nitric oxide (NO)-soluble GC and natriuretic peptide (NP)-particulate GC (pGC) pathways. Activation of these pathways has emerged as a potent therapeutic strategy to treat patients with heart failure, given cGMP-PKG signaling is impaired in heart failure with reduced ejection fraction (HFrEF) and preserved ejection fraction (HFpEF). Large scale clinical trials in patients with HFrEF have shown positive results with agents that activate cGMP-PKG pathways. In patients with HFpEF, however, benefits were observed only in a subgroup of patients. Further investigation for cGMP-PKG pathway is needed to develop better targeting strategies for HFpEF. This review outlines cGMP-PKG pathway and its modulation in heart failure.

## Introduction

Heart failure is a major health problem, and its prevalence is increasing worldwide. The traditional guideline directed therapies target the renin-angiotensin-aldosterone system and the sympathetic nervous system, but recently, cyclic guanosine 3′,5′-monophosphate (cGMP) and its downstream protein kinase G (PKG) signaling has attracted attention as a novel therapeutic target (Tsai and Kass, 2009). cGMP-PKG pathway regulates diverse cellular mechanisms to maintain cellular homeostasis and is activated by two different pathways. One is natriuretic peptide (NP)-NP receptor (NPR)-particulate guanylate cyclase (pGC) pathway, and the other is NO-soluble GC (sGC) pathway. cGMP-PKG pathway has been suggested to be blunted or dysregulated in patients with HFrEF or HFpEF (Paulus et al., 2013; Redfield et al., 2013). Increased plasma levels of inflammatory cytokines including TNF-α and IL-6 in HF are related to endothelial dysfunction with low NO-sGC-cGMP signaling in the heart and vasculature (Torre-Amione et al., 1996; Lommi et al., 1997), where its degradation by cGMP-PDEs might be enhanced. In HFpEF patients, myocardial homogenates from biopsy samples revealed low PKG activity and cGMP concentration compared with HFrEF and aortic stenosis patients (Van Heerebeek et al., 2012). Thus, the therapeutic strategy to recover blunted cGMP/PKG signaling in heart failure is very reasonable. Sacubitril/valsartan is the first agent in this class that has been approved for use in heart failure. It consists of the neprilysin (NEP) inhibitor and the angiotensin receptor blocker, and is described as an angiotensin receptor-neprilysin inhibitor (ARNi). NEP hydrolyzes several peptide hormones including NPs (ANP, BNP, CNP), adrenomedullin, glucagon, enkephalins, substance P, neurotensin, oxytocin, and bradykinin. Thus, its inhibition enhances NPs-pGC-cGMP. ARNi improved clinical outcomes in patients with HFrEF (McMurray et al., 2014; Velazquez et al., 2018) and also exhibited favorable outcomes in a particular sub-group (female) in HFpEF (Solomon et al., 2019; Pieske et al., 2021). Vericiguat, an sCG stimulator that enhances (NO)-sGC-cGMP pathway independently of NO, was approved for the treatment of heart failure. Vericugat is effective in patients with HFrEF (Armstrong et al., 2020a), but it failed to reveal clinical improvement in HFpEF (Armstrong et al., 2020b; Udelson et al., 2020) (Table 1). The clinical importance of cGMP-PKG pathway is clear; however, a better understanding of underlying mechanisms is necessary for the optimal therapeutic strategy with enhancement of cGMP-PKG signaling pathway. This review focuses on the regulatory mechanisms of cGMP-PKG pathway in heart failure.

**TABLE 1:** Clinical trials associated with sGC inhibitors and neprilysin inhibitors.

Study	Drugs	meanEF (%)	Number	Female (%)	NPs (pg/ml)	Outcomes	Notes
McMurray et al. (2014), PARADIGM-HF	Sacubitril- Valsartan (LCZ696)	29.6	4187	21.0	BNP 255 NT-proBNP 1631	A composite of death from CVD or hospitalization 21.8% vs 26.5% HR 0.80, 95% CI 0.73 to 0.87, *p* < 0.001	
	enalapril	29.4	4212	22.6	BNP 251 NT-proBNP 1594		
Velazquez et. al. (2019) PIONEER-HF	Sacubitril- Valsartan	24	440	25.7	NT-proBNP 4821	The time-averaged reduction in the NT-proBNP at weeks 4 and 8 to the baseline -46.7% vs -25.3%(ratio of change 0.76, 95% CI 0.69 to 0.85)	
	enalapril	25	441	30.2	4710		
Solomon et al. (2019) PARAGON-HF	Sacbitril- Vaslsartan	57.6	2419	51.6	NT-proBNP 904	Cardiovascular death 8.5% vs 8.9% HR 0.95, 95% CI 0.79 to 1.16	A composite outcome of hospitalization and cardiovascular death in female RR 0.73 95% CI 0.59 to 0.90
	Valsartan	57.5	2403	51.8	915	Total Hospitalization 690 vs 797 HR 0.85, 95% CI 0.72 to 1.0	
Pieske et al. (2021) PARALLAX	acbitril-Vaslsartan	56.7	1286	50.2	NT-proBNP 786	The reduction in NTproBNP at week 12 The adjusted geometric mean ratio 0.84 (95% CI, 0.80- 0.88; *p* < 0.001)	No significant between-group difference in the Kansas City Cardiomyopathy Questionnaire clinical summary score 12.3 vs 11.8 ( mean difference, 0.52; 95% CI, −0.93 to 1.97)
							No improvement in NYHA class 23.6% vs 24.0% of patients (adjusted odds ratio, 0.98; 95% CI, 0.81 to 1.18)
	Individualized comparator	56.2	1286	51.2	760	6-minute walk difference at week 24.	6-minute walking distance improved among women but decreased among men 6.59 vs −12.07 (*p* = 0.0024)
						No significant between-group from baseline 9.7 m vs 12.2 m	
						(adjusted mean difference, −2.5 m; 95% CI, −8.5 to 3.5; *p* = 0.42)	Individualized comparator: enalapril at a target dose of 10, valsartan at a target dose of 160 mg, or placebo (no RAS inhibitor).
Armstrong et al. (2020a) VICTORIA	Vericiguat	29.3	2526	24.0	NT-proBNP 2803	The composite of death from any cause or hospitalization for heart failure	
						37.9% vs 40.9%	
						HR 0.90, (95% CI 0.83 to 0.98, *p* = 0.02)	
	Placebo	27.9	2524	23.9	2821		
Udelson et al. (2020) CAPACITY HFpEF	Praliciguat	61.9	91	38.5	NT-proBNP 260	Changes in peak VO_2_	
						−0.26 vs −0.04 mL/kg/min	
						1286 (95% CI, −0.83 to 0.31 and –0.49 to 0.56)	
	Placebo	59.8	90	44.4	228.5		
Armstrong et al. (2020b) VITALITY-HFpEF	Vericiguat 15 mg	56.8	264	53.0	NT-proBNP 1364.5	The mean changes in the KCCQ PLS	The overall mortality rate was 4.1% (n = 32)
						5.5 points in the 15-mg/d vericiguat group	10 (3.8%) in the 15-mg vericiguat group
						6.5 points in the 10-mg/d vericiguat group	15 (5.7%) in the 10 mg vericiguat group
						6.9 points in the placebo group	7 (2.7%) in the placebo group
							8 cardiovascular deaths (3.0%) in the 15-mg vericiguat group
	Vericiguat 10mg	55.8	263	47.1	1339.1	differences between either vericiguat dosage and placebo were not statistically significant	12 (4.6%) in the 10-mg vericiguat group
	Placebo	56.3	262	46.2	1644.2		4 (1.5%) in the placebo group

### Phosphodiesterase and cGMP/PKG Signaling

Phosphodiesterase (PDE) has 11 superfamilies and more than 100 isoform variants that hydrolyze cAMP or cGMP to their inactive respective 5′-monophosphate form. Seven PDEs (PDE1, 2, 3, 4, 5, 8, and 9) are currently known to be expressed in myocardium. PDE1, 2, and 3 hydrolyze both cAMP and cGMP, while PDE5 and 9 are selective for cGMP and PDE4 and 8 are selective for cAMP (Kim and Kass, 2017). PDEs are differentially localized within the cells, contributing to the compartmentalized regulation of cGMP and cAMP signaling in both space and time.

Inhibition of PDE1, a dual substrate esterase, demonstrates acute inotropic and lusitropic effects largely via cAMP pathway (Hashimoto et al., 2018), demonstrated in large animal models. PDE1A, one the three isoforms of PDE1, modulates pathological hypertrophy via cGMP-PKG in rodent and cell models, while PDE1C, coupled with adenosine A2A receptor and TRPC3, hydrolyzes cAMP and regulates apoptosis in cardiac myocytes (Miller et al., 2009; Zhang et al., 2018).

PDE2 is also a dual substrate esterase and involved in the regulation of cardiac hypertrophy via cGMP. PDE2 specifically plays an important role in the crosstalk between cGMP and cAMP pathways because its activity is stimulated by cGMP (Baliga et al., 2018). PDE2 has three splice variants (PDE2A1, 2A2, 2A3), which are differently localized: PDE2A1 in cytoplasm, PDE2A2 in mitochondrial matrix, and PDE2A3 at membrane (mostly PDE2A3) (Geoffroy et al., 1999; Lugnier et al., 1999; Mongillo et al., 2006; Weber et al., 2017). In the heart, in particular, PDE2A might be localized in both cytosolic and particulate fractions of cardiac ventricle, though it differs from species to species (Le Trong et al., 1990; Bode et al., 1991; Muller et al., 1992; Sugioka et al., 1994; Geoffroy et al., 1999; Mongillo et al., 2006). In humans, PDE2A3 is expressed in cardiomyocytes and vascular endothelial cells (Sadhu et al., 1999). Under the normal conditions, PDE2 is less abundant in cardiomyocytes than in fibroblasts and endothelial cells (Stephenson et al., 2009; Vettel et al., 2014), but under the pathological conditions, PDE2 expressions and cAMP-hydrolyzing activity significantly increase (Levy, 2013; Mehel et al., 2013). (Chen et al., 2016). Cardiac PDE2A expressions increase in rat cardiac hypertrophy and also in human ischemic or non-ischemic heart failure (Mehel et al., 2013). PDE2 can hydrolyze cGMP produced by either pGC (Stangherlin et al., 2011) and sGC (Mongillo et al., 2006) with the allosteric hydrolyzing ability activated by cGMP, but this might depend on the stress conditions and cGMP concentrations (Terasaki and Appleman, 1975; Prigent et al., 1988; Mery et al., 1993; Dittrich et al., 2001; Herring et al., 2001; Weber et al., 2017). PDE2A overexpression blunts norepinephrine-induced cellular hypertrophy with marked decrease in cAMP levels (Mehel et al., 2013). On the other hand, PDE2A inhibition suppresses cardiac hypertrophy induced by norepinephrine in rats (Zoccarato et al., 2015). These apparently opposite results might be attributable to the cAMP and cGMP regulation levels which might depend on the contexts. In the computer modeling, Zhao et al. reported that PDE2A hydrolyzed increasing amount of cAMP with increasing levels of β adrenergic stimulation, and hydrolyzed increasing amounts of cGMP with decreasing levels of NO stimulation (Zhao et al., 2016).

**FIGURE 1:**
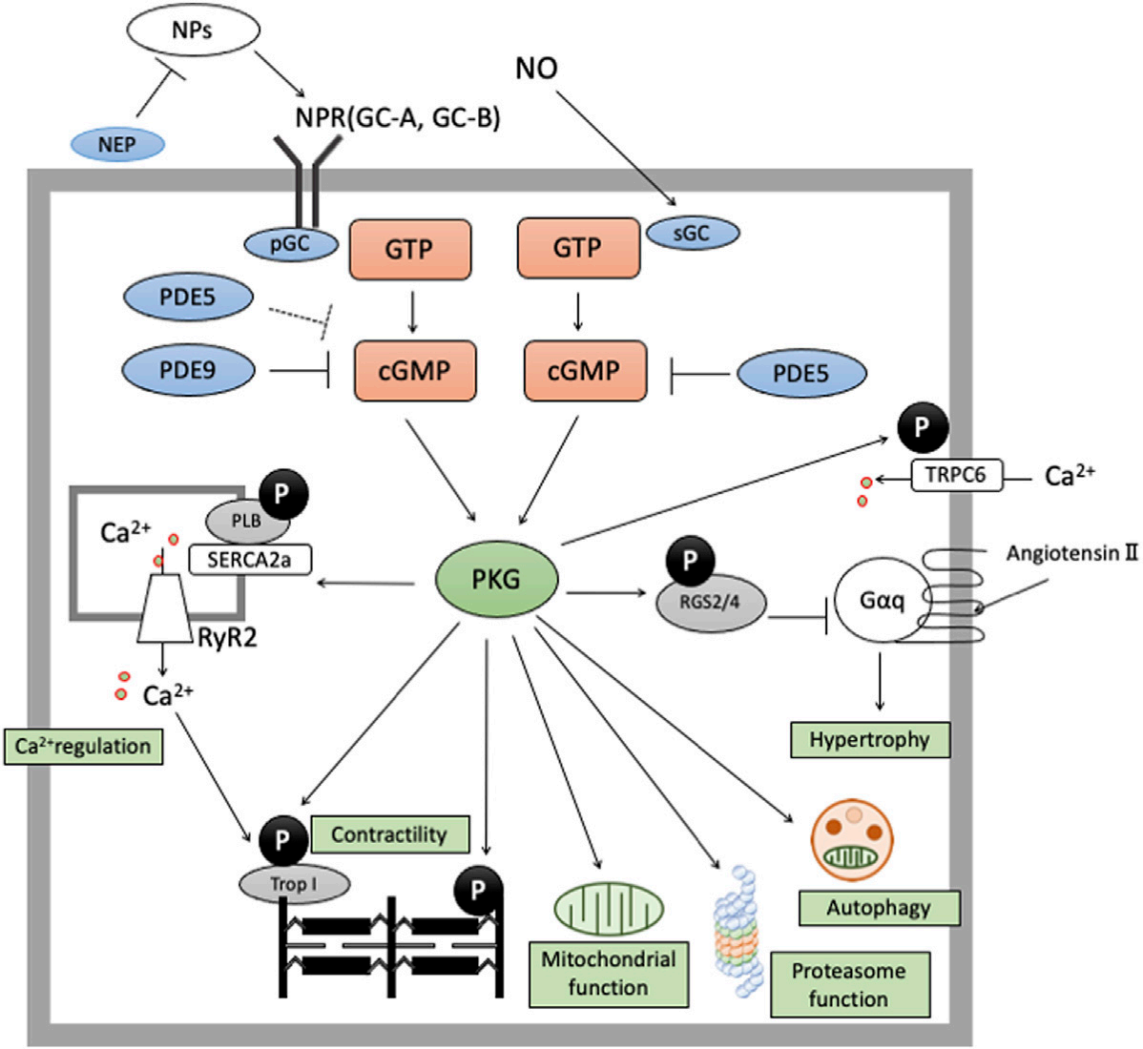
cGMP/PKG signaling in cardiomyocyte cGMP-PKG signaling is enhanced by two pathways. The former is NO-sGC-cGMP pathway and the latter is NP-NPR-pGC pathway. cGMP derived from NO-sGC pathway takes hydrolyzation by PDE5, and cGMP from NP-pGC pathway by PDE9 and PDE5 (especially in stressed conditions). cGMP/PKG signaling exerts protective effects in cardiomyocyte by phosphorylate various proteins like RGS2/4, Troponin I, TSC2, cMyBP-C, or Titin.

We elaborate on cGMP-specific PDEs (PDE5 and PDE9) in the next section, reviewing their effects on cardiac remodeling. PDE5 hydrolyzes cGMP derived from NO-sGC pathway and PDE9 degrades cGMP from NP-pGC pathway, modulating various signaling related to cardiac remodeling (Figure 1).

#### NO-sGC Pathway (PDE5)

Nitric oxide (NO) stimulates sGC to produce cGMP, which is hydrolyzed specifically by PDE5. PDE5A is localized at Z-disks in cardiac myocytes under physiological conditions but it is diffusely distributed under diseased conditions (Takimoto et al., 2005; Zhang et al., 2008). The expression of PDE5A is up-regulated in failing hearts (Shan et al., 2012), though it is very low under physiological conditions. In experimental animal models, PDE5 inhibition (PDE5i) provides cardiac protection against pressure-overload, ischemia-reperfusion injury, and doxorubicin-toxicity (Takimoto et al., 2005; Burley et al., 2007; Kukreja et al., 2012; Jin et al., 2013), with multiple myocardial signaling pathways altered (Takimoto, 2012). A regulator of G-protein signaling (RGS), 2/4 is phospho-activated to inhibit Gq-signaling (Takimoto et al., 2009) and transient receptor potential canonical Ca^2+^ channel-type6 (TRPC6) coupled with calcinurin (Cn) signaling (Koitabashi et al., 2010; Seo et al., 2014) is deactivated by PDE5i-PKG-phosphorylation. Mechanisms related to proteostasis are also regulated. PDE5i-activated PKG enhances proteasome function, blocking the accumulation of misfolded proteins via posttranslational modifications of proteasome subunits (Ranek et al., 2013). PDE5i also phosphorylates tuberin (TSC2), an intrinsic regulator of the mechanistic target of rapamycin complex-1 (mTORC1), and enhances autophagy. In a model of ischemia re-perfusion injury, PDE5i-cGMP-PKG exerts cardio-protective effects against necrosis and apoptosis through modulating mitochondrial functions (RAMZI et al., 2002; Salloum et al., 2008; Li et al., 2016; Patel et al., 2019) Additionally, PDE5i alone or in combination with natriuretic peptide, phosphorylates sarcomeric proteins including titin (Bishu et al., 2011a), troponin-I (Layland et al., 2005; Wijnker et al., 2014), and cardiac myosin-binding protein C (Thoonen et al., 2015), which improves systolic and diastolic function. Some may still have debate on PDE5A or PKG effects on cardiomyocyte. Straubinger et al. reported that sildenafil failed to limit the progressive cardiomyocyte growth, fibrosis, or cardiac dysfunctions in the cardiomyocyte-specific overexpression of the AT1 receptor mice (Straubinger et al., 2015). Lukowski et al. showed that the deletion of cardiomyocyte-specific PKG had no effect on cardiac hypertrophy caused by pressure overload and isoproterenol administration (Lukowski et al., 2010). Patrucco et al. reported that the lack of PKG in cardiomyocyte, endothelial cells, or cardiac fibroblast did not augment hypertrophic response and sildenafil had modest effects on angiotensin II-induced cardiac hypertrophy (Patrucco et al., 2014). On the other hand, however, cardiomyocyte-specific overexpression of PDE5A recovered impaired cardiac functions from pressure overload (Zhang et al., 2010) and myocardial infarction (Pokreisz et al., 2009). Frantz and Kuhn et al. generated animals with cardiomyocyte-restricted deletion of PKG, and demonstrated the animals developed severe hypertrophy by chronic angiotensin II infusion or pressure overload (Frantz et al., 2013). Recently, we have consistently reported that sildenafil exhibited protective effects against cardiac hypertrophy via proliferator activated receptor γ co-activator-1α-PKG cascade (Zhu et al., 2021). Together it would be reasonable to conclude that cGMP-PKG signaling in cardiomyocyte would be important in cardiac hypertrophy and remodeling. With regard to the cardiac-specific role and regulation of PDE5, a tissue-specific conditional deletion model would be awaited.

sGC stimulators and sGC activators are direct modulators of sGC, increasing the production of cGMP: the former stimulates NO-sensitive (unoxidized) sGC and the latter can activate NO-insensitive (oxidized) sGC. sGC stimulators have shown cardiac benefits in an HFpEF model (Wilck et al., 2018) as well as in an HFrEF model. Double-transgenic rats (dTGR) harboring the renin and angiotensinogen genes exhibit an HFpEF phenotype of diastolic dysfunction, preserved EF, systemic hypertension, cardiac hypertrophy, fibrosis, inflammation, and endothelial dysfunction, and dies between 7 and 8 weeks from severe heart failure (Damage et al., 1999; Mervaala et al., 2001; Wellner et al., 2005; Fischer et al., 2008; Finckenberg et al., 2012; Haase et al., 2014). Treatment with an sGC stimulator improved cardiac function, cardiac fibrosis, and inflammation, with minimal effects on cardiac hypertrophy (Wilck et al., 2018). sGC activators have also shown cardio-protective effects in another HFpEF model (Dahl salt-sensitive model: DSS) (Kolijn et al., 2020), where an sGC activator (cinaciguat) phosphorylates titin and improves passive stiffness. In human cardiomyocytes from HFpEF patients, cinaciguat phosphorylates titin and improves passive tension, associated with a reduction in proinflammatory cytokines and oxidative stress markers (Kolijn et al., 2020). sGC-bound cofactor heme (Fe2+) is oxidized to Fe^3+^ under oxidative conditions, leading to the inactive Apo form that no longer is responsive to NO. sGC stimulators stimulate only Fe^2+^-sGC, while sGC activators act on oxidated sGC(Fe^3+^-sGC or Apo-sGC) (Krishnan et al., 2018) to produce cGMP. In oxidated conditions such as HFpEF, sGC activator might have an advantage.

Although preclinical studies have revealed cardio-protective and anti-remodeling effects from NO-sGC-cGMP activation in either type of heart failure (HFrEF or HFpEF), clinical studies have yielded mixed results. Two meta-analyses of controlled clinical trials (928 patients in 14 studies (De Vecchis et al., 2017), 555 patients in 13 studies (De Vecchis et al., 2018)) demonstrate that PDE5 inhibitors improve clinical outcomes, exercise capacity, and pulmonary hemodynamics in patients with HFrEF, but not HFpEF. The negative results in HFpEF might be partially attributable to the female-specific response of PDE5i depending on estrogen levels, given the prevalence of HFpEF in older women: nearly half of the patients were older women (average age 67) in the negative RELAX trial. Epidemiological studies have demonstrated that women are likely to develop HFpEF. In clinical trials of HFpEF women account for around 50–60% of the trial cohorts (Forman and Gaziano, 2009; Savill, 2014), whereas they account for 20–25% of those of HFrEF (Pablo, 2017; Zannad et al., 2018; Pieske et al., 2019). In a recent multicenter, observational study, female sex was reported to be independently associated with the presence of diastolic dysfunction and worse clinical outcomes (Sotomi et al., 2021). Sex-hormone estrogen plays a pivotal role in cGMP-PKG signal coupled with NO via estrogen receptor (ERɑ)-mediated non-nuclear signaling, also known as rapid signaling or membrane-initiated steroid signaling (Adlanmerini et al., 2014; Arnal et al., 2017). In a female mouse model of heart failure, PDE5i fails to provide heart-protective effects in the absence of estrogen. We previously demonstrated that sildenafil treatment failed to exert anti-remodeling effects in female pathological hypertrophy heart in from Gαq-overexpressing or pressure-overloaded mice after ovary removal; on the contrary, estrogen replacement recovered the protective effects of sildenafil (Sasaki et al., 2014). Rüdebusch et al. (2020) also demonstrated that sGC stimulation has protective effects associated with improved gene expressions in mice heart failure model induced by pressure overload (Rüdebusch et al., 2020) and interestingly we have recently reported that this sGC protective effects are independent of estrogen status in rodent pressure-overload model (Nobuaki et al., 2020).

Despite promising preclinical results, however, a clinical study testing vericiguat in patients with HFpEF turned out negative (Vitality HFpEF). Although the reason for the negative results remains an open question, the redox status related to HFpEF might be speculated to be involved. NO–sGC–cGMP signaling can be compromised either by reducing the bioavailability of NO or by altering the redox state of sGC itself (Costell et al., 2012). Several groups reported that redox conditions altered cysteine residues (Cys) on sGC, affecting its catalytic or regulatory functions (Craven and DeRubertis, 1978a; Craven and DeRubertis, 1978b; Braughler, 1983). The redox status also alters the heme conditions within sGC. Heme iron in the reduced status (Fe^2+^) is necessary for NO binding, and sGC stimulator can stimulate only the reduced form of sGC, while the sGC activator can activate both reduced sGC and oxidized sGC (containing Fe^3+^) (Evgenov et al., 2006). In rat external iliac arteries without endothelium, peroxynitrite was reported to alter the redox state of sGC. Under the exposure of peroxynitrite, vascular relaxation induced by an sGC stimulator was impaired, whereas that by an sGC activator was enhanced. Additionally, this response correlated well with tissue levels of cGMP (Tawa et al., 2014). In Sprague Dawley rats fed with high salt/fat diet, an sGC activator, but not an sGC stimulator, attenuated the development of cardiac hypertrophy in a blood pressure-independent manner (Evgenov et al., 2006). Although there are no data about sGC redox status in patients with heart failure, inflammation and oxidative stress conditions in HFpEF might critically affect the efficacy of cGMP-modifying drugs (Tawa et al., 2014).

Thus, an sGC activator might serve as a potential novel treatment of HFpEF. So far, cinaciguat, an sGC activator, has been tested only in acute heart failure, with increased hypotensive events but no clear benefits, and sGC activators have not yet been explored in patients with chronic heart failure.

#### NP-pGC Pathway (PDE9 and PDE5)

Natriuretic peptides stimulate transmembrane receptor guanylate cyclase to produce cGMP. Atrial and B-type natriuretic peptides (ANP, BNP) bind to receptor particulate guanylyl cyclase A (pGC-A or NPRA), while C-type natriuretic peptide (CNP) binds to particulate guanylyl cyclase B (pGC-B or NPRB). pGC-A is localized at T-tubules and pGC-B is distributed throughout the sarcolemma. This spatial difference renders compartmentalized ANP/NPRA/cGMP signaling vs. CNP/NPRB/cGMP: the former have little impact on contractility and the latter have positive-lusitropic effects (Kuhn, 2016; Subramanian et al., 2018; Michel et al., 2020). cGMP from NP-pGC axis is degraded by PDE9 (Volpe et al., 2016; Goetze et al., 2020), which is expressed prominently in the brain and less in the heart (GraceKim et al., 2017). Similar to PDE5, myocardial PDE9 expression is low under physiological conditions but is upregulated under disease conditions such as HFpEF and aortic stenosis (Lee et al., 2015). PDE9 inhibition, either with a pharmacological or a genetic approach, suppressed cardiac hypertrophy in rodent pressure-overload (PO) model (Lee et al., 2015; Kokkonen-Simon et al., 2018; Richards et al., 2021). Importantly, both PDE5i and PDE9i similarly improve diastolic distensibility and ameliorate cardiac remodeling, associated with better profiles of hypertrophic/fibrosis-related gene expression (Lee et al., 2015), (Bishu et al., 2011a); however, comprehensive analyses of RNA-sequence data of myocardium reveals significant differences between PDE5i and PDE9i (Kokkonen-Simon et al., 2018), particularly in miRNA profiles related to hypertrophy and fibrosis: marked down-regulation of pro-hypertrophic and pro-fibrotic miRs by PDE5i vs. virtually no effect by PDE9i.

As previously described, ARNI exhibited favorable outcome in female patients with HFpEF (Solomon et al., 2019; Pieske et al., 2021). There has been no explanation provided for this observation of female-only benefit. We would speculate that this might be possibly related to difference of plasma NPs levels. Female patients with HFpEF are reported to exhibit lower plasma NPs levels as follows. ARNI might compensate lower levels of NPs in female patients with HFpEF. In HFpEF patients, plasma BNP levels are reported to be lower than in HFrEF (Harada et al., 2017); interestingly, women with HFpEF had lower BNP levels than men [43.9 vs. 76.1 pmol/L, *p* = 0.0193 (Tasevska-Dinevska et al., 2011), 104 vs 133, *p* < 0.001 (Savarese and D’Amario, 2018)] while in HFrEF the levels of NPs were inconsistent. One group reported that the plasma levels of ANP and BNP were similar in both genders (ANP: 114.9 vs. 141.2 pg/ml, *p* = 0.2606, BNP: 252.0 vs. 381.9 pg/ml, *p* = 0.1577). Another group reported that the plasma levels of NT-proBNP were higher in female HFrEF (8481 vs. 7543 pg/ml, *p* < 0.001) (Kim et al., 2017) and there is another group reporting that plasma NT-proBNP levels were similar in both genders (2532 vs. 2677 pg/ml, *p* = 0.978) (Sobhani et al., 2018). Another possible reason why ARNI is effective in female HFpEF might be related to CNP regulation. CNP exerts biological effects by binding to two types of natriuretic receptors; cGMP-coupled NPR-B and NPR-C (Chauhan et al., 2003; Villar et al., 2007). Endothelial deletion of CNP or global deletion of NPR-C revealed hypertensive phenotype only in female mice (Moyes et al., 2014), while the absence of eNOS and COX-1 in endothelial cells had no effect on mean blood pressure in female mice, but resulted in significantly high blood pressure in male animals (Scotland et al., 2005).

These suggest the pivotal contribution of CNP to female blood pressure maintenance. It is thus tempting to speculate that cardiac protection from ARNI therapy might depend more on the regulation of CNP in females than in males, although the contribution of cGMP might be unclear.

Although PDE5 hydrolyzes cGMP coupled with NO under normal conditions. PDE5 could become interactive with NPs-derived cGMP under stressed conditions (Zhang et al., 2012). Cardiomyocyte PDE5 is normally localized at Z-bands of sarcomeres, but becomes diffusely localized when exposed to pathological stress such as TAC or NOS inhibition (Nagayama et al., 2008; Zhang et al., 2012). In a dog hypertension model produced by bilateral renal wrapping, sildenafil treatment with concomitant BNP administration enhances plasma cGMP concentration, and recovers left ventricular diastolic capacitance in association with titin phosphorylation compared with sildenafil treatment alone (Bishu et al., 2011b). The beneficial synergistic effects of the combined PDE5 and NPs were also reported in pulmonary hypertension (PH). In a mouse model of hypoxia-induced PH, global deletion of NPRA blunts the beneficial effects of sildenafil on right ventricular systolic pressure (Zhao et al., 2003). Also, in hypoxia-induced PH rats, ANP and sildenafil show synergistic effects on decreasing right ventricular systolic pressure and on increasing plasma cGMP levels (Preston et al., 2004). Furthermore, a recent clinical trial of pulmonary arterial hypertension also demonstrated that the combined inhibition of neprilysin and PDE5 increase both plasma NP and cGMP levels and decreased pulmonary vascular resistance without affecting systemic blood pressure (Hobbs et al., 2019), which makes contrast to the concomitant use of PDE5 inhibitor (sildenafil) with sGC stimulator (riociguat) having been reported to be associated with hypotension but without beneficial effects on hemodynamics or exercise capacity (Galiè et al., 2015). The combination of pGC-related pathway and PDE5 might be a potential therapeutic option also in heart failure.

### PKG Oxidation in Failing Heart

**FIGURE 2:**
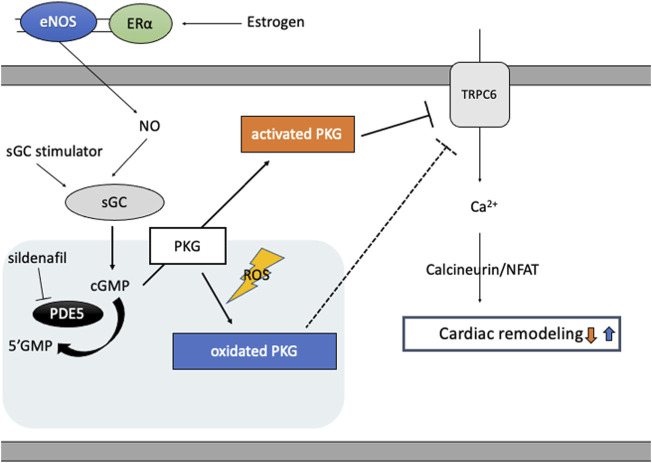
cGMP/PKG signaling and PKG oxidation. Estrogen plays a pivotal role in cGMP-PKG signaling coupled with eNOS via estrogen receptor (ERɑ)-mediated non-nuclear signaling. In diseased conditions like heart failure, eNOS activity is impaired and PKG undertakes oxidation and localizes in cytosol, inhibiting protective effects of PKG signaling independent of cGMP and enhancing cardiac remodeling via target proteins like TRPC6. PDE5 inhibitor, sildenafil, reveals protective effects only under the condition of sufficient oxidated PKG1α, while sGC stimulator improves cardiac remodeling independent of PKG redox status.

cGMP activated PKG targets various molecules to regulate cellular function in cardiomyocytes (Takimoto, 2012), including RGS2/4, TRPC6, proteasome systems, mitochondria, and sarcomere components. Two PKG genes, *prkg1* and *prkg2,* encode PKG1 and PKG2, respectively, and PKG1 is the primary isotype in cardiomyocyte. PKG1 is activated classically by cGMP, but also by oxidation (Figure 2): When oxidized, a cysteine residue C43(C42 in mice) forms a disulfide bond to form a homodimer of PKG1 (Burgoyne et al., 2007). Oxidized PKG1 is increased in failing hearts, though it accounts for only a small portion of PKG1 in normal hearts (Paulus et al., 2013; Nakamura et al., 2015; Prysyazhna et al., 2016). Oxidative PKG1 resides only at cytosol but not at the plasma membrane, while unoxidized PKG1 resides in both (Nakamura et al., 2015). Therefore, oxidized PKGI is no longer able to exert beneficial effects by the mechanisms mediated by membrane-localized PKGI, including inhibition of TRPC6-Cn-NFAT hypertrophy signaling and TSC2-mTORC1 metabolic/autophagy signaling (Oeing et al., 2020). Interestingly, PKG1 oxidation is required for the anti-remodeling effects from PDE5i as cytosol-localized PDE5 needs cGMP-activation via its GAF domain, while sGC stimulation exerts anti-remodeling effects independent of redox status of PKG1 (Nakamura et al., 2018). PDE5 inhibitor could be effective only under the sufficient myocardial stress to oxidate PKG1α, whereas an sGC stimulator provides benefits independent of redox conditions.

## Conclusion

cGMP/PKG signaling can be augmented by stimulation of either NO-sGC pathway or NP-pGC pathway. Although activation of either provides anti-remodeling benefits, they do not necessarily share the same molecular mechanisms in common. Furthermore, benefits might be also affected by the PKG redox status. Although ample preclinical evidence shows the benefits of cGMP/PKG augmentation in HFrEF or HFpEF models, clinical studies thus far provide consistent efficacy of cGMP/PKG augmentation in patients with HFrEF and limited efficacy in patients with HFpEF. Further studies would be helpful to better understand the pathophysiology of HFpEF and the development of novel treatments.

## References

[B1] Adlanmerini M. Solinhac R. Abot A. Fabre A. Raymond-Letron I. Guihot A.-L. (2014). Mutation of the Palmitoylation Site of Estrogen Receptor α *In Vivo* Reveals Tissue-specific Roles for Membrane versus Nuclear Actions. Proc. Natl. Acad. Sci. 111, E283 LP–E290. 10.1073/pnas.1322057111 24371309 PMC3896153

[B2] Armstrong P. W. Lam C. S. P. Anstrom K. J. Ezekowitz J. Hernandez A. F. O’Connor C. M. (2020). Effect of Vericiguat vs Placebo on Quality of Life in Patients with Heart Failure and Preserved Ejection Fraction: The VITALITY-HFpEF Randomized Clinical Trial. JAMA - J. Am. Med. Assoc. 324, 1512–1521. 10.1001/jama.2020.15922 PMC7576403 33079152

[B3] Armstrong P. W. Pieske B. Anstrom K. J. Ezekowitz J. Hernandez A. F. Butler J. (2020). Vericiguat in Patients with Heart Failure and Reduced Ejection Fraction. N. Engl. J. Med. 382, 1883–1893. 10.1056/NEJMoa1915928 32222134

[B4] Arnal J.-F. Lenfant F. Metivier R. Flouriot G. Henrion D. Adlanmerini M. (2017). Membrane and Nuclear Estrogen Receptor Alpha Actions: From Tissue Specificity to Medical Implications. Physiol. Rev. 97, 1045–1087. 10.1152/physrev.00024.2016 28539435

[B5] Baliga R. S. Preedy M. E. J. Dukinfield M. S. Chu S. M. Aubdool A. A. Bubb K. J. (2018). Phosphodiesterase 2 Inhibition Preferentially Promotes NO/guanylyl cyclase/cGMP Signaling to Reverse the Development of Heart Failure. Proc. Natl. Acad. Sci. U. S. A. 115, E7428–E7437. 10.1073/pnas.1800996115 30012589 PMC6077693

[B6] Bishu K. Hamdani N. Mohammed S. F. Kruger M. Ohtani T. Ogut O. (2011). Sildenafil and B-type Natriuretic Peptide Acutely Phosphorylate Titin and Improve Diastolic Distensibility *In Vivo* . Circulation 124, 2882–2891. 10.1161/CIRCULATIONAHA.111.048520 22144574 PMC3412357

[B7] Bishu K. Hamdani N. Mohammed S. F. Kruger M. Ohtani T. Ogut O. (2011). Sildenafil and B-type Natriuretic Peptide Acutely Phosphorylate Titin and Improve Diastolic Distensibility *In Vivo* . Circulation 124, 2882–2891. 10.1161/CIRCULATIONAHA.111.048520 22144574 PMC3412357

[B8] Bode D. C. Kanter J. R. Brunton L. L. (1991). Cellular Distribution of Phosphodiesterase Isoforms in Rat Cardiac Tissue. Circ. Res. 68, 1070–1079. 10.1161/01.res.68.4.1070 1849058

[B9] Braughler J. M. (1983). Soluble Guanylate Cyclase Activation by Nitric Oxide and its Reversal. Involvement of Sulfhydryl Group Oxidation and Reduction. Biochem. Pharmacol. 32, 811–818. 10.1016/0006-2952(83)90581-6 6132608

[B10] Burgoyne J. R. Madhani M. Cuello F. Charles R. L. Brennan J. P. Schröder E. (2007). Cysteine Redox Sensor in PKGIa Enables Oxidant-Induced Activation. Science 317, 13931397. 10.1126/science.1144318 17717153

[B11] Burley D. S. Ferdinandy P. Baxter G. F. (2007). Cyclic GMP and Protein Kinase-G in Myocardial Ischaemia-Reperfusion: Opportunities and Obstacles for Survival Signaling. Br. J. Pharmacol. 152, 855–869. 10.1038/sj.bjp.0707409 17700722 PMC2078226

[B12] Chauhan S. D. Nilsson H. Ahluwalia A. Hobbs A. J. (2003). Release of C-type Natriuretic Peptide Accounts for the Biological Activity of Endothelium-Derived Hyperpolarizing Factor. Proc. Natl. Acad. Sci. U. S. A. 100, 1426–1431. 10.1073/pnas.0336365100 12552127 PMC298789

[B13] Chen W. Spitzl A. Mathes D. Nikolaev V. O. Werner F. Weirather J. (2016). Endothelial Actions of ANP Enhance Myocardial Inflammatory Infiltration in the Early Phase after Acute Infarction. Circ. Res. 119, 237–248. 10.1161/CIRCRESAHA.115.307196 27142162

[B14] Costell M. H. Ancellin N. Bernard R. E. Zhao S. F. Upson J. J. Morgan L. A. (2012). Comparison of Soluble Guanylate Cyclase Stimulators and Activators in Models of Cardiovascular Disease Associated with Oxidative Stress. Front. Pharmacol. 3 (JUL), 1–14. 10.3389/fphar.2012.00128 22783192 PMC3389674

[B15] Craven P. A. DeRubertis F. R. (1978). Effects of Thiol Inhibitors on Hepatic Guanylate Cyclase Activity Evidence for the Involvement of Vicinal Dithiols in the Expression of Basal and Agonist-Stimulated Activity. BBA - Enzymol. 524, 231–244. 10.1016/0005-2744(78)90121-3 26412

[B16] Craven P. A. DeRubertis F. R. (1978). Restoration of the Responsiveness of Purified Guanylate Cyclase to Nitrosoguanidine, Nitric Oxide, and Related Activators by Heme and Hemeproteins. Evidence for Involvement of the Paramagnetic nitrosyl.Heme Complex in Enzyme Activation. J. Biol. Chem. 253, 8433–8443. 10.1016/s0021-9258(17)34310-7 30778

[B17] Damage H. E. Luft F. C. Mervaala E. Mu D. N. Gross V. Schmidt F. (1999). Influence of Exercise Training on Neurogenic Control of Blood Pressure in Spontaneously Hypertensive Rats. State-of-the-Art Lecture 34, 212–218. 10.1161/01.HYP.34.4.720 10523348

[B18] De Vecchis R. Cesaro A. Ariano C. (2018). Differential Effects of the Phosphodiesterase Inhibition in Chronic Heart Failure Depending on the Echocardiographic Phenotype (HFREF or HFpEF): a Meta-Analysis. Minerva Cardioangiol 66, 659–670. 10.23736/S0026-4725.17.04382-1 28398017

[B19] De Vecchis R. Cesaro A. Ariano C. Giasi A. Cioppa C. (2017). Phosphodiesterase-5 Inhibitors Improve Clinical Outcomes, Exercise Capacity and Pulmonary Hemodynamics in Patients with Heart Failure with Reduced Left Ventricular Ejection Fraction: A Meta-Analysis. J. Clin. Med. Res. 9, 488–498. 10.14740/jocmr3008w 28496549 PMC5412522

[B20] Dittrich M. Jurevicius J. Georget M. Rochais F. Fleischmann B. K. Hescheler J. (2001). Local Response of L-type Ca2+ Current to Nitric Oxide in Frog Ventricular Myocytes. J. Physiol. 534, 109–121. 10.1111/j.1469-7793.2001.00109.x 11432996 PMC2278687

[B21] Evgenov O. V. Pacher P. Schmidt P. M. Haskó G. Schmidt H. H. H. W. Stasch J. P. (2006). NO-independent Stimulators and Activators of Soluble Guanylate Cyclase: Discovery and Therapeutic Potential. Nat. Rev. Drug Discov. 5, 755–768. 10.1038/nrd2038 16955067 PMC2225477

[B22] Finckenberg P. Eriksson O. Baumann M. Merasto S. Lalowski M. M. Levijoki J. (2012). Caloric Restriction Ameliorates Angiotensin II-Induced Mitochondrial Remodeling and Cardiac Hypertrophy. Hypertension 59, 76–84. 10.1161/HYPERTENSIONAHA.111.179457 22068868

[B23] Fischer R. Dechend R. Qadri F. Markovic M. Feldt S. Herse F. (2008). Dietary N-3 Polyunsaturated Fatty Acids and Direct Renin Inhibition Improve Electrical Remodeling in a Model of High Human Renin Hypertension. Hypertension 51, 540–546. 10.1161/HYPERTENSIONAHA.107.103143 18158339

[B24] Forman D. Gaziano J. M. (2009). Irbesartan in Patients with Heart Failure and Preserved Ejection Fraction. Curr. Cardiovasc. Risk Rep. 3, 311–312. 10.1007/s12170-009-0056-1

[B25] Frantz S. Klaiber M. Baba H. A. Oberwinkler H. Völker K. Gaßner B. (2013). Stress-dependent Dilated Cardiomyopathy in Mice with Cardiomyocyte- Restricted Inactivation of Cyclic GMP-dependent Protein Kinase I. Eur. Heart J. 34, 1233–1244. 10.1093/eurheartj/ehr445 22199120 PMC3631523

[B26] Galiè N. Müller K. Scalise A. V. Grünig E. (2015). PATENT PLUS: A Blinded, Randomised and Extension Study of Riociguat Plus Sildenafil in Pulmonary Arterial Hypertension. Eur. Respir. J. 45, 1314–1322. 10.1183/09031936.00105914 25657022

[B27] Geoffroy V. Fouque F. Nivet V. Clot J. P. Lugnier C. Desbuquois B. (1999). Activation of a cGMP-Stimulated cAMP Phosphodiesterase by Protein Kinase C in a Liver Golgi-Endosomal Fraction. Eur. J. Biochem. 259, 892–900. 10.1046/j.1432-1327.1999.00123.x 10092879

[B28] Goetze J. P. Bruneau B. G. Ramos H. R. Ogawa T. de Bold M. K. de Bold A. J. (2020). Cardiac Natriuretic Peptides. Nat. Rev. Cardiol. 17, 698–717. 10.1038/s41569-020-0381-0 32444692

[B29] GraceKim E. PhDa David A. Kass M. (2017). Cardiac Phosphodiesterases and Their Modulation for Treating Heart Disease Grace. Handb. Exp. Pharmacol. 243, 249–269. 10.1007/164_2016_82 27787716 PMC5665023

[B30] Haase N. Rugor J. Przybyl L. Qadri F. Müller D. N. Dechend R. (2014). Relaxin Does Not Improve Angiotensin II-Induced Target-Organ Damage. PLoS One 9, 1–7. 10.1371/journal.pone.0093743 PMC3977876 24710077

[B31] Harada E. Mizuno Y. Kugimiya F. Shono M. Maeda H. Yano N. (2017). B-type Natriuretic Peptide in Heart Failure with Preserved Ejection Fraction: Relevance to Age-Related Left Ventricular Modeling in Japanese. Circ. J. 81, 1006–1013. 10.1253/circj.CJ-16-1282 28381705

[B32] Hashimoto T. Kim G. E. Tunin R. S. Adesiyun T. Hsu S. Nakagawa R. (2018). Acute Enhancement of Cardiac Function by Phosphodiesterase Type 1 Inhibition Translational Study in the Dog and Rabbit. Circulation 138, 1974–1987. 10.1161/CIRCULATIONAHA.117.030490 30030415 PMC6205901

[B33] Herring N. Rigg L. Terrar D. A. Paterson D. J. (2001). NO-cGMP Pathway Increases the Hyperpolarisation-Activated Current, if, and Heart Rate during Adrenergic Stimulation. Cardiovasc. Res. 52, 446–453. 10.1016/s0008-6363(01)00425-4 11738061

[B34] Hobbs A. J. Moyes A. J. Baliga R. S. Ghedia D. Ochiel R. Sylvestre Y. (2019). Neprilysin Inhibition for Pulmonary Arterial Hypertension: a Randomized, Double-Blind, Placebo-Controlled, Proof-Of-Concept Trial. Br. J. Pharmacol. 176, 1251–1267. 10.1111/bph.14621 30761523 PMC7651846

[B35] Jin Z. Zhang J. Zhi H. Hong B. Zhang S. Guo H. (2013). The Beneficial Effects of Tadalafil on Left Ventricular Dysfunction in Doxorubicin-Induced Cardiomyopathy. J. Cardiol. 62, 110–116. 10.1016/j.jjcc.2013.03.018 23731918

[B36] Kim G. E. Kass D. A. (2017). Cardiac Phosphodiesterases and Their Modulation for Treating Heart Disease. Handb. Exp. Pharmacol. 243, 249–269. 10.1007/164_2016_82 27787716 PMC5665023

[B37] Kim H. L. Kim M. A. Choi D. J. Han S. Jeon E. S. Cho M. C. (2017). Gender Difference in the Prognostic Value of N-Terminal Pro-B Type Natriuretic Peptide in Patients with Heart Failure ― a Report from the Korean Heart Failure Registry (KorHF). Circ. J. 81, 1329–1336. 10.1253/circj.CJ-16-1345 28442636

[B38] Koitabashi N. Aiba T. Hesketh G. G. Rowell J. Zhang M. Takimoto E. (2010). Cyclic GMP/PKG-dependent Inhibition of TRPC6 Channel Activity and Expression Negatively Regulates Cardiomyocyte NFAT Activation: Novel Mechanism of Cardiac Stress Modulation by PDE5 Inhibition. J. Mol. Cell. Cardiol. 48, 713–724. 10.1016/j.yjmcc.2009.11.015 19961855 PMC2837762

[B39] Kokkonen-Simon K. M. Saberi A. Nakamura T. Ranek M. J. Zhu G. Bedja D. (2018). Marked Disparity of microRNA Modulation by cGMP-Selective PDE5 versus PDE9 Inhibitors in Heart Disease. JCI Insight 3, e121739. 10.1172/jci.insight.121739 PMC6129132 30089721

[B40] Kolijn D. Kovács Á. Herwig M. Lódi M. Sieme M. Alhaj A. (2020). Enhanced Cardiomyocyte Function in Hypertensive Rats with Diastolic Dysfunction and Human Heart Failure Patients after Acute Treatment with Soluble Guanylyl Cyclase (sGC) Activator. Front. Physiol. 11, 1–21. 10.3389/fphys.2020.00345 32523538 PMC7261855

[B41] Krishnan S. M. Kraehling J. R. Eitner F. Bénardeau A. Sandner P. (2018). The Impact of the Nitric Oxide (No)/soluble Guanylyl Cyclase (sGC) Signaling cascade on Kidney Health and Disease: A Preclinical Perspective. Int. J. Mol. Sci. 19. 10.3390/ijms19061712 PMC6032334 29890734

[B42] Kuhn M. (2016). Molecular Physiology of Membrane Guanylyl Cyclase Receptors. Physiol. Rev. 96, 751–804. 10.1152/physrev.00022.2015 27030537

[B43] Kukreja R. C. Salloum F. N. Das A. (2012). Cyclic Guanosine Monophosphate Signaling and Phosphodiesterase-5 Inhibitors in Cardioprotection. J. Am. Coll. Cardiol. 59, 1921–1927. 10.1016/j.jacc.2011.09.086 22624832 PMC4230443

[B44] Layland J. Solaro R. J. Shah A. M. (2005). Regulation of Cardiac Contractile Function by Troponin I Phosphorylation. Cardiovasc. Res. 66, 12–21. 10.1016/j.cardiores.2004.12.022 15769444

[B45] Le Trong H. Walsh K. A. Charbonneau H. Beier N. Sonnenburg W. K. Stroop S. D. (1990). Amino Acid Sequence of the Cyclic GMP Stimulated Cyclic Nucleotide Phosphodiesterase from Bovine Heart. Biochemistry 29, 10280–10288. 10.1021/bi00496a018 2176866

[B46] Lee D. I. Zhu G. Sasaki T. Cho G.-S. Hamdani N. Holewinski R. (2015). Phosphodiesterase 9A Controls Nitric-oxide-independent cGMP and Hypertrophic Heart Disease. Nature 519, 472–476. 10.1038/nature14332 25799991 PMC4376609

[B47] Levy F. O. (2013). Cardiac PDEs and Crosstalk between cAMP and cGMP Signalling Pathways in the Regulation of Contractility. Naunyn. Schmiedebergs. Arch. Pharmacol. 386, 665–670. 10.1007/s00210-013-0874-z 23649864

[B48] Li N. Yuan Y. Li S. Zeng C. Yu W. Shen M. (2016). Pde5 Inhibitors Protect against post-infarction Heart Failure. Front. Biosci. - Landmark 21, 1194–1210. 10.2741/4450 27100500

[B49] Lommi J. Pulkki K. Koskinen P. Naveri H. Leinonen H. Harkonen M. (1997). Haemodynamic, Neuroendocrine and Metabolic Correlates of Circulating Cytokine Concentrations in Congestive Heart Failure. Eur. Heart J. 18, 1620–1625. 10.1093/oxfordjournals.eurheartj.a015142 9347273

[B50] Lugnier C. Keravis T. Le Bec A. Pauvert O. Proteau S. Rousseau E. (1999). Characterization of Cyclic Nucleotide Phosphodiesterase Isoforms Associated to Isolated Cardiac Nuclei. Biochim. Biophys. Acta - Gen. Subj. 1472, 431–446. 10.1016/s0304-4165(99)00145-2 10564757

[B51] Lukowski R. Rybalkin S. D. Loga F. Leiss V. Beavo J. A. Hofmann F. (2010). Cardiac Hypertrophy Is Not Amplified by Deletion of cGMP-dependent Protein Kinase I in Cardiomyocytes. Proc. Natl. Acad. Sci. U. S. A. 107, 5646–5651. 10.1073/pnas.1001360107 20212138 PMC2851748

[B52] McMurray J. J. V. Packer M. Desai A. S. Gong J. Lefkowitz M. P. Rizkala A. R. (2014). Angiotensin–Neprilysin Inhibition versus Enalapril in Heart Failure. N. Engl. J. Med. 371, 993–1004. 10.1056/NEJMoa1409077 25176015

[B53] Mehel H. Emons J. Vettel C. Wittköpper K. Seppelt D. Dewenter M. (2013). Phosphodiesterase-2 Is Up-Regulated in Human Failing Hearts and Blunts β-adrenergic Responses in Cardiomyocytes. J. Am. Coll. Cardiol. 62, 1596–1606. 10.1016/j.jacc.2013.05.057 23810893

[B54] Mervaala E. M. A. Cheng Z. J. Tikkanen I. Lapatto R. Nurminen K. Vapaatalo H. (2001). Endothelial Dysfunction and Xanthine Oxidoreductase Activity in Rats with Human Renin and Angiotensinogen Genes. Hypertension 37, 414–418. 10.1161/01.hyp.37.2.414 11230310

[B55] Mery P. Pavoines C. Belhassen L. Peckers F. (1993). Nitric Oxide Regulates Cardiac Ca2+ Current. J. Biol. Chem. 268, 26286–26295. 10.1016/s0021-9258(19)74313-0 7902837

[B56] Michel K. Herwig M. Werner F. Spes K. Š. Abeßer M. Schuh K. (2020). C-type Natriuretic Peptide Moderates Titin-Based Cardiomyocyte Stiffness. JCI Insight 5. 10.1172/jci.insight.139910 PMC7710274 33055420

[B57] Miller C. L. Oikawa M. Cai Y. Wojtovich A. P. Nagel D. J. Xu X. (2009). Role of Ca2+/calmodulin-Stimulated Cyclic Nucleotide Phosphodiesterase 1 in Mediating Cardiomyocyte Hypertrophy. Circ. Res. 105, 956–964. 10.1161/CIRCRESAHA.109.198515 19797176 PMC2803071

[B58] Mongillo M. Tocchetti C. G. Terrin A. Lissandron V. Cheung Y. F. Dostmann W. R. (2006). Compartmentalized Phosphodiesterase-2 Activity Blunts β-adrenergic Cardiac Inotropy via an NO/cGMP-dependent Pathway. Circ. Res. 98, 226–234. 10.1161/01.RES.0000200178.34179.93 16357307

[B59] Moyes A. J. Khambata R. S. Villar I. Bubb K. J. Baliga R. S. Lumsden N. G. (2014). Endothelial C-type Natriuretic Peptide Maintains Vascular Homeostasis. J. Clin. Invest. 124, 4039–4051. 10.1172/JCI74281 25105365 PMC4151218

[B60] Muller B. Stoclet J. C. Lugnier C. (1992). Cytosolic and Membrane-Bound Cyclic Nucleotide Phosphodiesterases from guinea Pig Cardiac Ventricles. Eur. J. Pharmacol. Mol. Pharmacol. 225, 263–272. 10.1016/0922-4106(92)90028-t 1325367

[B61] Nagayama T. Zhang M. Hsu S. Takimoto E. Kass D. A. (2008). Sustained Soluble Guanylate Cyclase Stimulation Offsets Nitric-Oxide Synthase Inhibition to Restore Acute Cardiac Modulation by Sildenafil. J. Pharmacol. Exp. Ther. 326, 380–387. 10.1124/jpet.108.137422 18456872

[B62] Nakamura T. Ranek M. J. Lee D. I. Hahn V. S. Kim C. Eaton P. (2015). Prevention of PKG1a Oxidation Augments Cardioprotection in the Stressed Heart. J. Clin. Invest. 125, 2468–2472. 10.1172/JCI80275 25938783 PMC4497760

[B63] Nakamura T. Zhu G. Ranek M. J. Kokkonen-Simon K. Zhang M. Kim G. E. (2018). Prevention of PKG-1α Oxidation Suppresses Antihypertrophic/Antifibrotic Effects from PDE5 Inhibition but Not sGC Stimulation. Circ. Hear. Fail. 11, e004740. 10.1161/CIRCHEARTFAILURE.117.004740 PMC5858464 29545395

[B64] Nobuaki F. Eiki T. Kazutaka U. Pangyen L. Miyu T. Yu O. (2020). Estrogen Receptor-α Non-nuclear Signaling Confers Cardioprotection and Is Essential to cGMP-PDE5 Inhibition Efficacy. JACC Basic Transl. Sci. 5, 282–295. 32215350 10.1016/j.jacbts.2019.12.009 PMC7091505

[B65] Oeing C. U. Nakamura T. Pan S. Mishra S. Dunkerly-Eyring B. L. Kokkonen-Simon K. M. (2020). PKG1a Cysteine-42 Redox State Controls mTORC1 Activation in Pathological Cardiac Hypertrophy. Circ. Res. 127, 522–533. 10.1161/CIRCRESAHA.119.315714 32393148 PMC7416445

[B66] Pablo A. (2017). Olavegogeascoechea. De la evidencia a la práctica en la insuficiencia cardíaca. Rev. Argentina Med. 5, 132–133.

[B67] Patel C. H. Nakamura T. Zhu G. Bedja D. Sasaki M. Holewinski R. J. (2019). PKG1-modified TSC2 Regulates mTORC1 Activity to Counter Adverse Cardiac Stress. Nature 566, 264–269. 30700906 10.1038/s41586-019-0895-y PMC6426636

[B68] Patrucco E. Domes K. Sbroggió M. Blaich A. Schlossmann J. Desch M. (2014). Roles of cGMP-dependent Protein Kinase I (cGKI) and PDE5 in the Regulation of Ang II-Induced Cardiac Hypertrophy and Fibrosis. Proc. Natl. Acad. Sci. U. S. A. 111, 12925–12929. 10.1073/pnas.1414364111 25139994 PMC4156763

[B69] Paulus W. J. Tschöpe C. D P. H. (2013). A Novel Paradigm for Heart Failure with Preserved Ejection Fraction: Comorbidities Drive Myocardial Dysfunction and Remodeling through Coronary Microvascular Endothelial Inflammation. J. Am. Coll. Cardiol. 62, 263–271. 10.1016/j.jacc.2013.02.092 23684677

[B70] Pieske B. Patel M. J. Westerhout C. M. Anstrom K. J. Butler J. Ezekowitz J. (2019). Baseline Features of the VICTORIA (Vericiguat Global Study in Subjects with Heart Failure with Reduced Ejection Fraction) Trial. Eur. J. Heart Fail. 21, 1596–1604. 10.1002/ejhf.1664 31820546

[B71] Pieske B. Wachter R. Shah S. J. Baldridge A. Szeczoedy P. Ibram G. (2021). Effect of Sacubitril/Valsartan vs Standard Medical Therapies on Plasma NT-proBNP Concentration and Submaximal Exercise Capacity in Patients with Heart Failure and Preserved Ejection Fraction: The PARALLAX Randomized Clinical Trial. JAMA - J. Am. Med. Assoc. 326, 1919–1929. 10.1001/jama.2021.18463 PMC8596197 34783839

[B72] Pokreisz P. Vandenwijngaert S. Bito V. Van Bergh A. Den. Lenaerts I. Busch C. (2009). Ventricular Phosphodiesterase-5 Expression Is Increased in Patients with Advanced Heart Failure and Contributes to Adverse Ventricular Remodeling after Myocardial Infarction in Mice. Circulation 119, 408–416. 10.1161/CIRCULATIONAHA.108.822072 19139381 PMC3791110

[B73] Preston I. R. Hill N. S. Gambardella L. S. Warburton R. R. Klinger J. R. (2004). Synergistic Effects of ANP and Sildenafil on cGMP Levels and Amelioration of Acute Hypoxic Pulmonary Hypertension. Exp. Biol. Med. 229, 920–925. 10.1177/153537020422900908 15388887

[B74] Prigent A. F. Fougier S. Nemoz G. Anker G. Pacheco H. Lugnier C. (1988). Comparison of Cyclic Nucleotide Phosphodiesterase Isoforms from Rat Heart and Bovine Aorta. Separation and Inhibition by Selective Reference Phosphodiesterase Inhibitors. Biochem. Pharmacol. 37, 3671–3681. 10.1016/0006-2952(88)90400-5 2845994

[B75] Prysyazhna O. Burgoyne J. R. Scotcher J. Grover S. Kass D. Eaton P. (2016). Phosphodiesterase 5 Inhibition Limits Doxorubicin-Induced Heart Failure by Attenuating Protein Kinase G Iα Oxidation. J. Biol. Chem. 291, 17427–17436. 10.1074/jbc.M116.724070 27342776 PMC5016139

[B76] Ramzi O. Salloum F. Hawkins J. Kukreja R. C. (2002). Sildenafil (Viagra) Induces Powerful Cardioprotective Effect via Opening of Mitochondrial KATP Channels in Rabbits. Am. J. Physiol. Hear. Circ. Physiol. 283, H1263–H1269. 10.1152/ajpheart.00324.2002 12181158

[B77] Ranek M. J. Terpstra E. J. M. Li J. Kass D. A. Wang X. (2013). Protein Kinase G Positively Regulates Proteasome-Mediated Degradation of Misfolded Proteins. Circulation 128, 365–376. 10.1161/CIRCULATIONAHA.113.001971 23770744 PMC3761383

[B78] Redfield M. M. Chen H. H. Borlaug B. A. Semigran M. J. Lee K. L. Lewis G. (2013). Effect of Phosphodiesterase-5 Inhibition on Exercise Capacity and Clinical Status in Heart Failure with Preserved Ejection Fraction: A Randomized Clinical Trial. JAMA - J. Am. Med. Assoc. 309, 1268–1277. 10.1001/jama.2013.2024 PMC3835156 23478662

[B79] Richards D. A. Aronovitz M. J. Liu P. Martin G. L. Tam K. Pande S. (2021). CRD-733, a Novel PDE9 (Phosphodiesterase 9) Inhibitor, Reverses Pressure Overload-Induced Heart Failure. Circ. Hear. Fail. 14, e007300. 10.1161/circheartfailure.120.007300 PMC8451972 33464954

[B80] Rüdebusch J. Benkner A. Nath N. Fleuch L. Kaderali L. Grube K. (2020). Stimulation of Soluble Guanylyl Cyclase (sGC) by Riociguat Attenuates Heart Failure and Pathological Cardiac Remodelling. Br. J. Pharmacol. 1, 13. 10.1111/bph.15333 33247945

[B81] Sadhu K. Hensley K. Florio V. A. Wolda S. L. (1999). Differential Expression of the Cyclic GMP-Stimulated Phosphodiesterase PDE2A in Human Venous and Capillary Endothelial Cells. J. Histochem. Cytochem. 47, 895–905. 10.1177/002215549904700707 10375378

[B82] Salloum F. N. Abbate A. Das A. Houser J. E. Mudrick C. A. Qureshi I. Z. (2008). Sildenafil (Viagra) Attenuates Ischemic Cardiomyopathy and Improves Left Ventricular Function in Mice. Am. J. Physiol. - Hear. Circ. Physiol. 294, 1398–1406. 10.1152/ajpheart.91438.2007 18223185

[B83] Sasaki H. Nagayama T. Blanton R. M. Seo K. Zhang M. Zhu G. (2014). PDE5 Inhibitor Efficacy Is Estrogen Dependent in Female Heart Disease. J. Clin. Invest. 124, 2464–2471. 10.1172/JCI70731 24837433 PMC4089449

[B84] Savarese G. D’Amario D. (2018). Sex Differences in Heart Failure. Adv. Exp. Med. Biol. 1065, 529–544. 10.1007/978-3-319-77932-4_32 30051405

[B85] Savill P. (2014). Spironolactone in Heart Failure with Preserved Ejection Fraction. Practitioner 258, 10.

[B86] Scotland R. S. Madhani M. Chauhan S. Moncada S. Andresen J. Nilsson H. (2005). Investigation of Vascular Responses in Endothelial Nitric Oxide Synthase/cyclooxygenase-1 Double-Knockout Mice: Key Role for Endothelium-Derived Hyperpolarizing Factor in the Regulation of Blood Pressure *In Vivo* . Circulation 111, 796–803. 10.1161/01.CIR.0000155238.70797.4E 15699263

[B87] Seo K. Rainer P. P. Lee D. I. Hao S. Bedja D. Birnbaumer L. (2014). Hyperactive Adverse Mechanical Stress Responses in Dystrophic Heart Are Coupled to Transient Receptor Potential Canonical 6 and Blocked by Cgmp-Protein Kinase G Modulation. Circ. Res. 114, 823–832. 10.1161/CIRCRESAHA.114.302614 24449818 PMC3963144

[B88] Shan X. Quaile M. P. Monk J. K. French B. Cappola T. P. Margulies K. B. (2012). Differential Expression of Pde5 in Failing and Nonfailing Human Myocardium. Circ. Hear. Fail. 5, 79–86. 10.1161/CIRCHEARTFAILURE.111.961706 PMC3261338 22135403

[B89] Sobhani K. Nieves Castro D. K. Fu Q. Gottlieb R. A. Van Eyk J. E. Noel Bairey Merz C. (2018). Sex Differences in Ischemic Heart Disease and Heart Failure Biomarkers. Biol. Sex. Differ. 9, 1–13. 10.1186/s13293-018-0201-y 30223899 PMC6142320

[B90] Solomon S. D. McMurray J. J. V. Anand I. S. Ge J. Lam C. S. P. Maggioni A. P. (2019). Angiotensin–Neprilysin Inhibition in Heart Failure with Preserved Ejection Fraction. N. Engl. J. Med. 381, 1609–1620. 10.1056/NEJMoa1908655 31475794

[B91] Sotomi Y. Hikoso S. Nakatani D. Mizuno H. Okada K. Dohi T. (2021). Sex Differences in Heart Failure with Preserved Ejection Fraction. J. Am. Heart Assoc. 10, 1–20. 10.1161/jaha.120.018574 PMC8174270 33619973

[B92] Stangherlin A. Gesellchen F. Zoccarato A. Terrin A. Fields L. A. Berrera M. (2011). CGMP Signals Modulate Camp Levels in a Compartment-specific Manner to Regulate Catecholamine-dependent Signaling in Cardiac Myocytes. Circ. Res. 108, 929–939. 10.1161/CIRCRESAHA.110.230698 21330599 PMC3083836

[B93] Stephenson D. T. Coskran T. M. Wilhelms M. B. Adamowicz W. O. O’Donnell M. M. Muravnick K. B. (2009). Immunohistochemical Localization of Phosphodiesterase 2A in Multiple Mammalian Species. J. Histochem. Cytochem. 57, 933–949. 10.1369/jhc.2009.953471 19506089 PMC2746727

[B94] Straubinger J. Schöttle V. Bork N. Subramanian H. Dünnes S. Russwurm M. (2015). Sildenafil Does Not Prevent Heart Hypertrophy and Fibrosis Induced by Cardiomyocyte Angiotensin II Type 1 Receptor Signalings. J. Pharmacol. Exp. Ther. 354, 406–416. 10.1124/jpet.115.226092 26157043

[B95] Subramanian H. Froese A. Jönsson P. Schmidt H. Gorelik J. Nikolaev V. O. (2018). Distinct Submembrane Localisation Compartmentalises Cardiac NPR1 and NPR2 Signalling to cGMP. Nat. Commun. 9, 1–9. 10.1038/s41467-018-04891-5 29934640 PMC6014982

[B96] Sugioka M. Ito M. Masuoka H. Ichikawa K. Konishi T. Tanaka T. (1994). Identification and Characterization of Isoenzymes of Cyclic Nucleotide Phosphodiesterase in Human Kidney and Heart, and the Effects of New Cardiotonic Agents on These Isoenzymes. Naunyn. Schmiedebergs. Arch. Pharmacol. 350, 284–293. 10.1007/BF00175034 7824045

[B97] Takimoto E. (2012). Cyclic GMP-dependent Signaling in Cardiac Myocytes. Circ. J. 76, 1819–1825. 10.1253/circj.cj-12-0664 22785374

[B98] Takimoto E. Koitabashi N. Hsu S. Ketner E. A. Zhang M. Nagayama T. (2009). The. Regulator of G Protein Signaling 2 Mediates Cardiac Compensation to Pressure Overload and Antihypertrophic Effects of PDE5 Inhibition in Mice. J. Clin. Invest. 119, 408–420. 10.1172/JCI35620 19127022 PMC2631292

[B99] Takimoto E. Champion H. C. Li M. Belardi D. Ren S. Rodriguez E. R. (2005). Chronic Inhibition of Cyclic GMP Phosphodiesterase 5A Prevents and Reverses Cardiac Hypertrophy. Nat. Med. 11, 214–222. 10.1038/nm1175 15665834

[B100] Tasevska-Dinevska G. Kennedy L. M. Cline-Iwarson A. Cline C. Erhardt L. Willenheimer R. (2011). Gender Differences in Variables Related to B-Natriuretic Peptide, Left Ventricular Ejection Fraction and Mass, and Peak Oxygen Consumption, in Patients with Heart Failure. Int. J. Cardiol. 149, 364–371. 10.1016/j.ijcard.2010.02.018 20202706

[B101] Tawa M. Shimosato T. Iwasaki H. Imamura T. Okamura T. (2014). Effects of Peroxynitrite on Relaxation through the NO/sGC/cGMP Pathway in Isolated Rat Iliac Arteries. J. Vasc. Res. 51, 439–446. 10.1159/000371491 25634663

[B102] Terasaki W. L. Appleman M. M. (1975). The Role of Cyclic GMP in the Regulation of Cyclic AMP Hydrolysis. Metabolism 24, 311–319. 10.1016/0026-0495(75)90112-2 165353

[B103] Thoonen R. Giovanni S. Govindan S. Lee D. I. Wang G.-R. Calamaras T. D. (2015). Molecular Screen Identifies Cardiac Myosin-Binding Protein-C as a Protein Kinase G-Iα Substrate. Circ. Heart Fail. 8, 1115–1122. 10.1161/CIRCHEARTFAILURE.115.002308 26477830 PMC4651836

[B104] Torre-Amione G. Kapadia S. Benedict C. Oral H. Young J. B. Mann D. L. (1996). Proinflammatory Cytokine Levels in Patients with Depressed Left Ventricular Ejection Fraction: A Report from the Studies of Left Ventricular Dysfunction (SOLVD). J. Am. Coll. Cardiol. 27, 1201–1206. 10.1016/0735-1097(95)00589-7 8609343

[B105] Tsai E. J. Kass D. A. (2009). Cyclic GMP Signaling in Cardiovascular Pathophysiology and Therapeutics. Pharmacol. Ther. 122, 216–238. 10.1016/j.pharmthera.2009.02.009 19306895 PMC2709600

[B106] Udelson J. E. Lewis G. D. Shah S. J. Zile M. R. Redfield M. M. Burnett J. (2020). Effect of Praliciguat on Peak Rate of Oxygen Consumption in Patients with Heart Failure with Preserved Ejection Fraction: The CAPACITY HFpEF Randomized Clinical Trial. JAMA - J. Am. Med. Assoc. 324, 1522–1531. 10.1001/jama.2020.16641 PMC7576408 33079154

[B107] Van Heerebeek L. Hamdani N. Falcão-Pires I. Leite-Moreira A. F. Begieneman M. P. V. Bronzwaer J. G. F. (2012). Low Myocardial Protein Kinase G Activity in Heart Failure with Preserved Ejection Fraction. Circulation 126, 830–839. 10.1161/CIRCULATIONAHA.111.076075 22806632

[B108] Velazquez E. J. Morrow D. A. DeVore A. D. Duffy C. I. Ambrosy A. P. McCague K. (2018). Angiotensin–Neprilysin Inhibition in Acute Decompensated Heart Failure. N. Engl. J. Med. 380, 539–548. 10.1056/NEJMoa1812851 30415601

[B109] Vettel C. Lämmle S. Ewens S. Cervirgen C. Emons J. Ongherth A. (2014). PDE2-mediated cAMP Hydrolysis Accelerates Cardiac Fibroblast to Myofibroblast Conversion and Is Antagonized by Exogenous Activation of cGMP Signaling Pathways. Am. J. Physiol. - Hear. Circ. Physiol. 306, 1246–1252. 10.1152/ajpheart.00852.2013 24531807

[B110] Villar I. C. Panayiotou C. M. Sheraz A. Madhani M. Scotland R. S. Nobles M. (2007). Definitive Role for Natriuretic Peptide Receptor-C in Mediating the Vasorelaxant Activity of C-type Natriuretic Peptide and Endothelium-Derived Hyperpolarising Factor. Cardiovasc. Res. 74, 515–525. 10.1016/j.cardiores.2007.02.032 17391657 PMC3503309

[B111] Volpe M. Carnovali M. Mastromarino V. (2016). The Natriuretic Peptides System in the Pathophysiology of Heart Failure: From Molecular Basis to Treatment. Clin. Sci. 130, 57–77. 10.1042/CS20150469 PMC5233571 26637405

[B112] Weber S. Zeller M. Guan K. Wunder F. Wagner M. El-Armouche A. (2017). PDE2 at the Crossway between cAMP and cGMP Signalling in the Heart. Cell. Signal. 38, 76–84. 10.1016/j.cellsig.2017.06.020 28668721

[B113] Wellner M. Dechend R. Park J. K. Shagdarsuren E. Al-Saadi N. Kirsch T. (2005). Cardiac Gene Expression Profile in Rats with Terminal Heart Failure and Cachexia. Physiol. Genomics 20, 256–267. 10.1152/physiolgenomics.00165.2004 15623567

[B114] Wijnker P. J. M. Murphy A. M. Stienen G. J. M. van der Velden (2014). J. Troponin I Phosphorylation in Human Myocardium in Health and Disease. Neth. Heart J. 22, 463–469. 10.1007/s12471-014-0590-4 25200323 PMC4188840

[B115] Wilck N. Markó L. Balogh A. Kräker K. Herse F. Bartolomaeus H. (2018). Nitric Oxide-Sensitive Guanylyl Cyclase Stimulation Improves Experimental Heart Failure with Preserved Ejection Fraction. JCI insight 3. 10.1172/jci.insight.96006 PMC5916255 29467337

[B116] Zannad F. Anker S. D. Byra W. M. Cleland J. G. F. Fu M. Gheorghiade M. (2018). Rivaroxaban in Patients with Heart Failure, Sinus Rhythm, and Coronary Disease. N. Engl. J. Med. 379, 1332–1342. 10.1056/NEJMoa1808848 30146935

[B117] Zhang M. Koitabashi N. Nagayama T. Rambaran R. Feng N. Takimoto E. (2008). Expression, Activity, and Pro-hypertrophic Effects of PDE5A in Cardiac Myocytes. Cell. Signal. 20, 2231–2236. 10.1016/j.cellsig.2008.08.012 18790048 PMC2601628

[B118] Zhang M. Takimoto E. Hsu S. Lee D. I. Nagayama T. Danner T. (2010). Myocardial Remodeling Is Controlled by Myocyte-Targeted Gene Regulation of Phosphodiesterase Type 5. J. Am. Coll. Cardiol. 56, 2021–2030. 10.1016/j.jacc.2010.08.612 20970280 PMC3036840

[B119] Zhang M. Takimoto E. Lee D. I. Santos C. X. C. Nakamura T. Hsu S. (2012). Pathological Cardiac Hypertrophy Alters Intracellular Targeting of Phosphodiesterase Type 5 from Nitric Oxide Synthase-3 to Natriuretic Peptide Signaling. Circulation 126, 942–951. 10.1161/CIRCULATIONAHA.112.090977 22829024 PMC3428375

[B120] Zhang Y. Knight W. Chen S. Mohan A. Yan C. (2018). Multiprotein Complex with TRPC (Transient Receptor Potential-Canonical) Channel, PDE1c (Phosphodiesterase 1C), and A2R (Adenosine A2 Receptor) Plays a Critical Role in Regulating Cardiomyocyte cAMP and Survival. Circulation 138, 1988–2002. 10.1161/CIRCULATIONAHA.118.034189 29871977 PMC6205915

[B121] Zhao C. Y. Greenstein J. L. Winslow R. L. (2016). Roles of Phosphodiesterases in the Regulation of the Cardiac Cyclic Nucleotide Cross-Talk Signaling Network. J. Mol. Cell. Cardiol. 91, 215–227. 10.1016/j.yjmcc.2016.01.004 26773602 PMC4764497

[B122] Zhao L. Mason N. A. Strange J. W. Walker H. Wilkins M. R. (2003). Beneficial Effects of Phosphodiesterase 5 Inhibition in Pulmonary Hypertension Are Influenced by Natriuretic Peptide Activity. Circulation 107, 234–237. 10.1161/01.cir.0000050653.10758.6b 12538421

[B123] Zhu G. Ueda K. Hashimoto M. Zhang M. Sasaki M. Kariya T. (2021). The Mitochondrial Regulator PGC1α Is Induced by cGMP–PKG Signaling and Mediates the Protective Effects of Phosphodiesterase 5 Inhibition in Heart Failure. FEBS Lett. 596, 17–28. 10.1002/1873-3468.14228 34778969 PMC9199229

[B124] Zoccarato A. Surdo N. C. Aronsen J. M. Fields L. A. Mancuso L. Dodoni G. (2015). Cardiac Hypertrophy Is Inhibited by a Local Pool of cAMP Regulated by Phosphodiesterase 2. Circ. Res. 117, 707–719. 10.1161/CIRCRESAHA.114.305892 26243800

